# Development and initial validation of the Cardiovascular Disease Acceptance and Action Questionnaire (CVD-AAQ) in an Italian sample of cardiac patients

**DOI:** 10.3389/fpsyg.2014.01284

**Published:** 2014-11-14

**Authors:** Chiara A. M. Spatola, Emanuele A. M. Cappella, Christina L. Goodwin, Matteo Baruffi, Gabriella Malfatto, Mario Facchini, Gianluca Castelnuovo, Gian Mauro Manzoni, Enrico Molinari

**Affiliations:** ^1^Psychology Research Laboratory, Istituto Auxologico Italiano – Istituto di Ricovero e Cura a Carattere ScientificoMilan, Italy; ^2^Department of Psychology, Catholic University of MilanMilan, Italy; ^3^The Ohio State UniversityColumbus, OH, USA; ^4^Cardiology Division, Istituto Auxologico Italiano – Istituto di Ricovero e Cura a Carattere ScientificoMilan, Italy; ^5^Faculty of Psychology, eCampus UniversityNovedrate, Como, Italy

**Keywords:** psychological inflexibility, acceptance, cardiovascular disease, adherence to treatment, experiential avoidance, Cardiovascular Disease Acceptance and Action Questionnaire

## Abstract

Psychological inflexibility refers to the attempt to decrease internal distress even when doing so is inconsistent with life values, and has been identified as a potential barrier to making and maintaining health behavior changes that are consistent with a heart-healthy lifestyle. Disease- and behavior-specific measures of psychological inflexibility have been developed and utilized in treatment research. However, no specific measure has been created for patients with heart disease. Thus, the CardioVascular Disease Acceptance and Action Questionnaire (CVD-AAQ) was developed. The present study is aimed to evaluate the psychometric properties of the CVD-AAQ and to explore its association with measures of psychological adjustment and cardiovascular risk factors in an Italian sample of 275 cardiac patients. Exploratory factor analysis showed a structural one-factor solution with satisfactory internal consistency and test–retest reliability. The relation with other measures was in the expected direction with stronger correlations for the theoretically consistent variables, supporting convergent and divergent validity. CVD-AAQ scores were associated with general psychological inflexibility, anxiety and depression and inversely correlated with psychological well-being. Moreover, the results showed that CVD-AAQ scores are associated with two relevant risk factors for cardiac patients, namely low adherence to medication and being overweight. In sum, results suggest that the CVD-AAQ is a reliable and valid measure of heart disease-specific psychological inflexibility with interesting clinical applications for secondary prevention care.

## INTRODUCTION

A large portion of cardiovascular risk can be attributed to lifestyle factors, such as unhealthy diet, low physical activity, and smoking (Yusuf et al., 2004). Although the reduction of these unhealthy habits has resulted in a significant decline in mortality rate comparable to that shown by cardio-preventive pharmacotherapy, few patients successfully improve their negative health-behaviors and even fewer maintain acquired changes in the long-term (Dorneleas, 2008). Moreover, high non-adherence rates to cardio-protective medications and subsequent adverse outcomes have been well documented (Ho et al., 2008). Current research has demonstrated that difficulties with behavior change may be related to a lack of psychological flexibility, a concept targeted in acceptance and commitment therapy (ACT; Hayes et al., 1999).

Psychological flexibility is defined as the ability to be fully present in each moment which includes accepting one’s own internal experiences even when unpleasant and distressful, and willingly engaging in behaviors that are consistent with one’s life values (Hayes et al., 2006).

Two major concepts are involved in the definition of psychological flexibility: the present moment contact with one’s thoughts, emotions, and bodily sensations (experiential acceptance), and the importance of maintaining values-driven behaviors. In contrast, psychological *in*flexibility is the attempt to decrease cognitive and physical distress even when doing so may sacrifice long-term behavioral goals (Luoma et al., 2011).

Since adopting and maintaining a healthy lifestyle often entails distressing internal experiences, individuals with a low ability to tolerate internal distress might be less likely to adopt and maintain behavioral changes. Previous research evaluating adherence to health-behaviors note the importance of distress tolerance; for example, changes in diet often require compromises in portion-size and taste (Forman et al., 2007), exercise is associated with physical discomfort and exercise-anxiety (Butryn et al., 2011), and abstaining from nicotine is associated with cravings (Gifford et al., 2004; Brown et al., 2005). The relevant role of psychological inflexibility in preventing behavior change is confirmed by recent studies, showing the promise of acceptance-based interventions at increasing physical activity (Butryn et al., 2011), the ability of managing food cravings (Forman et al., 2007), and weight loss maintenance (Forman et al., 2009).

Moreover, variables found to be impacted by psychological inflexibility, such as depression, anxiety and stress have been shown to negatively impact cardiovascular risk and prognosis (Januzzi et al., 2000; Rozanski et al., 2005; Dimsdale, 2008; Rothenbacher et al., 2014) through the mediation of biological mechanisms such as hypertension and blood pressure reactivity to stress, (Strike and Steptoe, 2004) and behavioral pathways such as lower medication adherence and lesser likelihood to adopt healthy behaviors (Rothenbacher et al., 2014).

Psychological inflexibility is also inversely associated with quality of life, perceived health, and positive emotional experiences (Bond et al., 2011; Gaudiano, 2011). Thus, it is important to investigate the relationship between psychological inflexibility, mental health, quality of life, and the development and progression of coronary heart disease. Understanding such associations will be especially useful in the context of secondary prevention efforts, such as cardiac rehabilitation.

While general measures of psychological inflexibility do exist, to date no instrument has been validated for reliably measuring cardiovascular disease-specific psychological inflexibility. The most established measure of general psychological flexibility is the Acceptance and Action Questionnaire (AAQ; Hayes et al., 2004), which cuts across diagnostic categories and psychological problems. Research on psychological inflexibility in many different samples has shown that the AAQ scores predict 16–25% of the variance in outcomes across a broad range of psychological health conditions (Hayes et al., 2006). However, versions with a specific focus may offer greater precision in the measurement of psychological flexibility within particular contexts or populations. For example, acceptance of pain as measured by the Chronic Pain Acceptance Questionnaire (CPAQ) appears to partially mediate the relationship between pain severity, and the degree of pain interference and emotional distress (Fish et al., 2010). Similarly, body image flexibility improved prediction of disordered eating, above and beyond overall psychological flexibility even after controlling for body image dissatisfaction and BMI (Sandoz et al., 2013). Comparable results have been shown for other specific variants of the AAQ and related measures of psychological flexibility in such areas as obesity (Lillis et al., 2009), diabetes (Gregg et al., 2007), epilepsy (Lundgren et al., 2008), tinnitus (Westin et al., 2008), substance abuse (Luoma et al., 2011) and smoking (Gifford et al., 2004).

These results provide support for the usefulness of developing measures of psychological flexibility that are domain specific.

Cardiovascular disease is a disease that requires adjustment to both change in lifestyle and change in self-conception (i.e., identification as a cardiovascular patient). This change in self-conception can be difficult and potentially harmful if the person with heart disease becomes distressed as a result of this new self-conception and begins to avoid disease-specific thoughts, feelings, and behaviors. Behavior-specific measures related to the adoption of healthy lifestyles, such as the Food AAQ (Juarascio et al., 2011) or the Physical Activity AAQ (Butryn et al., 2014) could be usefully adopted with cardiac patients. However, their use will provide information about those behaviors independent of heart disease, which is less helpful when attempting to alter behavior in the context of cardiovascular disease and a changing self-conception. Lastly, a single disease-specific measure will require less time from patients and participants than multiple behavior-specific measures, thus reducing overall patient burden.

Therefore the CardioVascular Disease AAQ (CVD-AAQ) was created to assess patient tendency to avoid difficult disease-related thoughts and feelings and the consequent lack of willingness to engage in recommended life-style changes.

The aim of the present study is threefold:

(1) to evaluate the psychometric properties of CVD-AAQ;
(2) to explore the association between patient demographics and CVD-AAQ scores;
(3) to evaluate the association between CVD-AAQ scores, rates of medication adherence and BMI in a sample of cardiac patients.

## MATERIALS AND METHODS

### PARTICIPANTS

This study involved 275 participants who were receiving outpatient cardiac rehabilitation.

Demographic information is summarized in **Table 1**. The majority of participants were married, completed high school, and retired from the workforce.

**Table 1:** Demographic characteristics of the sample.

Demographic variables
Mean age (years)	65.50
Gender (% female)	21.80
**Marital status (%)**
Never married	10.20
Married	68.70
Separated	5.10
Divorced	5.10
Widowed	10.90
**Education (%)**
Elementary school	18.20
Junior high school	25.50
High school	40.70
College education	15.60
**Employment (%)**
Employed	32.00
Retired	57.50
Housewives	5.10
Unemployed	5.50

### MEASURES

#### General and disease-specific psychological infexibility

The *CVD-AAQ* is an adaptation of a more general measure of experiential acceptance and avoidance, the AAQ (Bond et al., 2011). The CVD-AAQ was created to measure individual acceptance of thoughts and feelings related to cardiovascular illness and the degree to which such thoughts and feelings interfere with valued action. The CVD-AAQ items were developed by two of the authors (CAMS and CLG) who had prior experience with both third generation cognitive behavioral treatments and cardiac rehabilitation patients. The CVD-AAQ initial items pool contained 10-items (e.g., I avoid thinking about the risks I face if I don’t take care of my heart) rated on a 7-point Likert-type scale ranging from 1 (never true) to 7 (always true). High scores represent high psychological inflexibility.

General psychological inflexibility was measured using the *AAQ-II* (Bond et al., 2011) a seven-item measure of psychological inflexibility and experiential avoidance. High scores represent more psychological inflexibility. The Italian version of the questionnaire has shown good reliability (α = 0.83) and high convergent validity (Pennato et al., 2013). Cronbach’s alpha for the current study is 0.75.

#### General psychological well-being

Psychological health was measured by the *Psychological General Well-Being (PGWB) Inventory* (Dupuy, 1984), validated in Italian (Grossi et al., 2002). Six affective states are assessed within six subscales: anxiety, depressed mood, positive well-being, self-control, general health, and vitality. For each subscale higher scores reflect higher levels of well-being. In the current study PGWB Cronbach’s alpha was 0.94 for the total score, 0.86 for anxiety, 0.83 for depressed mood, 0.67 for self-control, 0.83 for positive well-being, 0.59 for general health, and 0.81 for vitality.

#### Psychological stress

Psychological stress was measured using the 10-item *Perceived Stress Scale*, (PSS-10; Cohen et al., 1983), assessing the degree to which situations in one’s life are appraised as stressful. For the current study, the scale was translated to Italian. The PSS-10 has shown good validity and internal reliability ranging from 0.78 to 0.91 (Cohen and Janicki-Deverts, 2012). Cronbach’s alpha for this study is 0.83.

#### Self-efficacy

The *Generalized Self-Efficacy Scale* (GSE; Schwarzer and Jerusalem, 1995), was utilized as a measure of self-efficacy. It assesses one’s sense of personal competence and ability to complete tasks and reach goals, even when facing stressful situations (Bandura, 1977). The Italian version of the questionnaire has shown acceptable levels of reliability (α = 0.79; Scholz et al., 2002). Cronbach’s alpha for the current study was 0.88.

#### Anxiety and depression

Anxiety and depression scores were assessed using the *Hospital Anxiety and Depression Scale* (HADS), a 14-item self-report scale (Zigmond and Snaith, 1983) comprised of an anxiety and a depression subscale. Higher scores indicate more depression and anxiety. The Italian version of the scale has shown good validity and high internal consistency (α = 0.89; Costantini et al., 1999). Cronbach’s alpha for the current sample ranged from 0.48–0.56.

#### Coping strategies

Coping strategies were measured using the *Brief-COPE* (Carver, 1997), an instrument designed to evaluate how people handle stressful events. The Italian version of the scale has been validated (Conti, 1999) and includes 14 coping reactions: active coping, planning, positive reframing, acceptance, humor, religion, using emotional support, using instrumental support, self-distraction, denial, venting, substance use, behavioral disengagement, and self-blame. The alpha reliabilities of this 14 subscales ranged from 0.50 to 0.90 (Carver, 1997). In the present sample four scales (self-blame, venting, behavioral disengagement, substance use) showed unsatisfactory reliability with an alpha value lower than 0.50, and were thus excluded from subsequent analyses. The alpha values of the remaining subscales ranged from 0.51 (for Active Coping) to 0.80 (for Religion).

#### Adherence to medication

Adherence to pharmacological treatment was measured by the *Morisky Medical Adherence Scale-4 items* (MMAS-4; Morisky et al., 1986). Higher scores indicate less adherence. The MMAS-4 showed moderate reliability (α = 0.61) and good criterion-related, concurrent, and predictive validity (Morisky et al., 1986). In the present study Cronbach’s alpha was 0.60.

### PROCEDURE

Data were collected in the context of the ACTonHEART study, whose protocol was approved by the Istituto Auxologico Italiano Ethics Committee. Participants were recruited over a 16-month period by research staff at the beginning of the Cardiac Rehabilitation program. After giving permission to the use of their data for research purposes, participants completed a battery of measures including a demographics questionnaire, the HADS, the BRIEF-COPE, the CVD-AAQ item pool, the Morisky Adherence Scale, the GSE, the AAQ-II, and the PGWB in that order. A subgroup of participants (*n* = 132) also completed the PSS. Additional information such as weight, height and smoking status were self-reported by all participants. In order to evaluate test–retest reliability the CVD-AAQ was re-administered to 216 participants at one of three time points: after 7–14 days (*n* = 57), after 21–28 days (*n* = 110), and after 35–42 days (*n* = 49).

## RESULTS

### EXPLORATORY FACTOR ANALYSIS

A exploratory factor analysis (EFA) was conducted on the initial 10-item version of the questionnaire. The sampling adequacy for the analysis, verified by the Kaiser–Meyer–Olkin (KMO) index was very good for the total scale (KMO = 0.81) and acceptable for all individual items (KMO ≥ 0.57). Bartlett’s test of sphericity indicated that correlations between items were sufficiently large for EFA [χ^2^(45) = 592.66, *p* < 0.0001].

An initial analysis was conducted with the principal axis factoring method with no restriction to the number of factors to be estimated. The initial run resulted in two factors with eigenvalues over Kaiser criterion of 1 (Kaiser, 1974), namely 3.22 and 1.56, explaining 47.86% of the total variance. The scree plot depicted a point of inflexion that would justify retaining two factors, with a third factor barely under 1 (0.942).

To better understand what model best represented the data, a parallel analysis (Horn, 1965) was computed. Previous research indicates that parallel analysis is a more accurate factor extraction procedure (Zwick and Velicer, 1986) than the scree plot or Kaiser’s criterion. The results of the parallel analysis confirmed a two-factor model.

Since the two factors were expected to be elements of a single higher-order structure, namely psychological flexibility, an oblique rotation (Direct Oblimin) was applied (Nunnally, 1978). However, the percentage of variance explained by the two factors did not improve after rotation, and the correlation between the two factors remained very low (*r* = 0.04).

Moreover, reliability analyses indicated that items comprising the second factor (items 1, 2, 6) did not significantly correlate with the overall scale and their removal significantly increased Cronbach’s alpha. An EFA of the remaining seven items was computed to evaluate the impact of item deletion to the scale’s structure.

The scree plot depicted a point of inflection that suggested retaining one factor. This result was in line with the analysis of eigenvalues, with only one over Kaiser’s criterion of 1 (3.12), explaining 44.55% of the variance. Thus, a one-factor solution was deemed appropriate, with a single factor thought to represent psychological inflexibility. In order to further justify a one-factor solution a second parallel analysis (Horn, 1965) was conducted. This additional analysis confirmed a one-factor solution for our measure. **Table 2** shows the factor loadings from principal axis factoring of one and two-factor solutions, the latter without oblique rotation.

**Table 2:** Factor loadings from principal axis factoring.

CVD-AAQ item	Two-factor solution		One-factor solution
(r0.3pcl.1pc)2-3	Factor 1	Factor 2	
1. I try to avoid thinking about having heart disease		0.47	/
2. I can lead a full and meaningful life even with heart disease^a^		0.47	/
3. I do not take care of my health as I should in order to avoid thinking I have heart disease	0.66		0.67
4. I eat things that are dangerous for my heart because the urge to eat them is overwhelming	0.39		0.39
5. Thinking about my heart disease is too or stressful for me	0.61		0.60
6. When I have an upsetting feeling or thought about my heart disease I try to get rid of it		0.57	/
7. I avoid taking or forget to take my medication because it reminds me that I have heart disease	0.57		0.58
8. I do not exercise regularly because it reminds me that I have heart disease	0.64		0.65
9. I avoid thinking about the risks I face if I don’t take care of my heart	0.60		0.60
10. I feel so scared by the thought of a possible relapse that I am not able to commit myself to what really matters in my life	0.67		0.65
% of variance	32.20	15.66	44.55
α		0.67	0.79

*Only loadings above 0.3 are presented in the table.*

*^a^Item reversed for scoring purposes.*

The internal consistency of the 7-item scale was found to be satisfactory, with a Cronbach’s alpha of 0.79. All item-total correlations were above 0.50 except for item 4 (*r* = 0.36). These reliability indices were much improved from the 10-item version, which showed a Cronbach’s alpha of 0.67 and a lower item-total correlation (item 1, 2, and 6 below 0.30). Items 1, 2, and 6 were deleted and subsequent analyses utilized the 7-item version of the CVD-AAQ.

### TEST-RETEST RELIABILITY

This section of the study was designed to examine test–retest reliability by calculating the two-way mixed, absolute concordance intraclass correlation coefficient (ICC). Of the 216 participants who completed the CVD-AAQ for a second time, a first subsample (*n* = 57) was retested after 7–14 days, a second group (*n* = 110) after 21–28 days, and a third group (*n* = 49) after 35–42 days. The three groups did not significantly differ concerning demographics such as age [*F*(2,216) = 1.62, *p* = 0.20], gender [χ^2^(2) = 0.32, *p* = 0.85], employment status [χ^2^(6) = 6.65, *p* = 0.35], marital status [χ^2^(8) = 7.33, *p* = 0.50], and education [χ^2^(6) = 3.38, *p* = 0.76]. Moreover the three groups did not significantly differ concerning CVD-AAQ scores at time 1 [*F*(2,216) = 1.94, *p* = 0.15].

Mean CVD-AAQ scores did not significantly change from the first to second administration for all three gathered together [*t*(235) = 0.19; *p* = 0.85]. Test–retest reliability appeared to decrease over time. According to Fleiss (1986), it was excellent after 7–14 days (ICC = 0.90; CI = 0.83–0.94), good after 21–28 days (ICC = 0.82; CI = 0.73–0.87), but only fair after 35–42 days (ICC = 0.66; CI = 0.40–0.81).

### RELATIONSHIP WITH DEMOGRAPHICS

A univariate analysis of variance (ANOVA) was conducted to explore the impact of gender, age, marital status, education, and employment status on psychological inflexibility related to cardiac disease.

As expected, there were no significant differences between CVD-AAQ scores in relation to gender [*F*(1) = 0.39, *p* = 0.53], age [*F*(1) = 2.68, *p* = 0.10], marital status [*F*(4) = 0.35, *p* = 0.84], education [*F*(3) = 2.07, *p* = 0.10] and employment status [*F*(3) = 1.98, *p* = 0.12].

### VALIDITY

#### Construct validity

In order to assess convergent validity a Pearson correlation coefficient was calculated between CVD-AAQ and AAQ-II. If the CVD-AAQ measures psychological inflexibility specific to the cardiovascular disease, it would be expected to be moderately correlated with measures of general psychological inflexibility. As shown in **Table 3**, the correlation between CVD-AAQ scores and AAQ-II (*r* = 0.50; *p* < 0.001) supported the hypothesized relationship. Such correlation was stronger than the association with all the other measures, further supporting the construct validity of CVD-AAQ.

**Table 3:** Correlations between CVD-AAQ and other measures.

	CVD-AAQ	
	*r*	*p*
AAQ-II	0.50	<0.001
HADS sum score	0.45	<0.001
HADS anxiety	0.44	<0.001
HADS depression	0.34	<0.001
PGWB anxiety	0.39	<0.001
PGWB depressed mood	0.45	<0.001
PGWB self-control	-0.44	<0.001
PGWB positive well-being	-0.35	<0.001
PGWB general health	-0.37	<0.001
PGWB vitality	-0.41	<0.001
PGWB sum score	-0.48	<0.001
PSS	0.44	<0.001
GSE	-0.25	<0.001
Brief-Cope planning	-0.10	0.01
Brief-Cope self-distraction	0.14	0.02
Brief-Cope using instrumental support	-0.02	0.72
Brief-Cope using emotional support	0.13	0.04
Brief-Cope positive reframing	-0.06	0.30
Brief-Cope acceptance	-0.20	0.001
Brief-Cope religion	-0.07	0.26
Brief-Cope humor	-0.01	0.85
Brief-Cope denial	0.22	<0.001

*Positive correlations reflect higher levels of inflexibility and distress. AAQ-II, Acceptance and Action Questionnaire-II; HADS, Hospital Anxiety and Depression Scale; PGWB, Psychological General Well Being; PSS, Perceived Stress Scale; GSE, General Self-Efficacy Scale.*

The associations of the CVD-AAQ with the different coping strategies assessed by the BRIEF-COPE were in line with our expectations. Cardiac-specific psychological inflexibility appeared to be significantly correlated with the “denial” subscale and to be inversely correlated with the “acceptance” scale. Although the association is weak, these results are consistent with the conceptual relatedness of these two scales with the experiential avoidance construct. Moreover, the remaining BRIEF-COPE scales did not show significant correlations with CVD-AAQ, further supporting the divergent validity of the instrument.

#### Concurrent validity

As expected, CVD-AAQ scores were significantly correlated with other psychological constructs of interest (see **Table 3**). In particular, higher levels of heart disease-specific psychological inflexibility were significantly and moderately related to higher anxiety and depression (on both the HADS and the PGWB), higher stress (measured by the PSS), and to lower psychological well-being (on both the general health and vitality dimensions of PGWB). Moreover, a weak but significant correlation was found with self-efficacy (GSE).

Lastly, CVD-AAQ was significantly correlated with problems on adherence to medication (*r* = 0.27; *p* < 0.001) and BMI (*r* = 0.23; *p* < 0.001), while general psychological inflexibility, measured by the AAQ-II, reported a weaker association with the adherence measure (*r* = 0.15; *p* = 0.01) and no correlation with BMI (*r* = 0.06; *p* = 0.33).

#### Independent sample *t*-test

A two-tailed independent samples *t*-test showed that overweight participants (i.e., BMI > 25) had significantly higher CVD-AAQ scores (*M* = 17.61, SD = 7.00), than those with a BMI below 25 [*M* = 15.00, SD = 5.90; *t*(272) = 3.29, *p* < 0.001] with a small effect size (*r* = 0.20). On the contrary, overweight (*M* = 19.53, SD = 8.50) and non-overweight (*M* = 18.31, SD = 7.50) patients did not significantly differ with respect to AAQ-II scores [*t*(301) = 1.30, *p* < 0.19].

An independent samples *t*-test was then conducted to compare the CVD-AAQ score for patients with good adherence to medication (Morisky score = 0) and patients with low adherence (Morisky score ≥ 1). This latter category includes a wide range of patients with a suboptimal level of medication adherence ranging from occasionally missing a dose to missing the majority of medication doses. However, the above classification was considered clinically relevant, and was thus utilized for these analyses.

Patients with low adherence (*M* = 18.02, SD = 6.72) showed significantly higher scores on CVD-AAQ than patients with good medication adherence [*M* = 15.27, SD = 6.20; *t*(263) = 3.34, *p* < 0.001] with a small effect size (*r* = 0.19). This finding was not replicated with the AAQ-II; participants with low adherence, (*M* = 19.90, SD = 7.45) did not differ significantly on scores of general psychological inflexibility [*t*(285) = -1.38, *p* < 0.17] from those with good medication adherence (*M* = 18.51, SD = 8.38).

## DISCUSSION

Although psychological inflexibility has been reported to play an important role in health-behavior change, there is currently no measure of this construct in the context of heart disease. Therefore, the CVD-AAQ was developed to fill the existing gap in the literature. The current study sought to investigate the psychometric characteristics of the CVD-AAQ in terms of factorial structure, reliability and validity.

The initial item pool was generated by applying the psychological inflexibility construct to the context of cardiovascular disease in order to measure the extent to which an individual makes cognitive and behavioral choices that are consistent with his/her values of health even when doing so lead to experiencing distressing thoughts or feelings. An EFA on this 10-item scale suggested a two-factor solution. However, reliability analyses showed that items loadings on the second factor did not significantly correlate with the overall scale and their deletion significantly increased Cronbach’s alpha. After the items’ removal, the resulting seven-item scale showed a 1-factor structure, a solution consistent with the AAQ-II. In conclusion, CVD-AAQ appears to measure the construct of psychological inflexibility with satisfactory internal consistency and test–retest reliability.

In addition to its sound factor structure and good reliability, our findings indicate that the CVD-AAQ is associated with variables to which it is theoretically tied. Specifically, the greatest association was observed with the AAQ-II, a general measure of the same construct, supporting the convergent validity of the instrument. On the other side, the absence of a significant correlation with all the coping strategies measured by the BRIEF-COPE, except for denial and acceptance, supported the divergent validity of the instrument.

Moreover, higher levels of cardiac-specific psychological inflexibility were concurrently associated with greater depressive symptoms, anxiety, and psychological stress and lower levels of psychological well-being. These results are highly consistent with previous research on general psychological inflexibility, which appeared to be a key process in the etiology and maintenance of psychopathology and psychological suffering (Hayes et al., 2006; Gaudiano, 2011).

Consistent with the existing literature (Bond et al., 2011), psychological inflexibility was not associated with demographic variables. Cardiac-specific psychological inflexibility was greater in patients with low rates of adherence to medical treatment—findings that mirror previous research on disease-specific psychological inflexibility (Weijman et al., 2005). This finding suggests that psychological flexibility may be associated with the adoption and maintenance of health behaviors, such as medication adherence, among patients with heart disease.

Cardiovascular Disease Acceptance and Action Questionnaire yields substantially different scores among cardiac patients with a BMI >25 compared with those with a BMI below 25. This finding indicates that the overweight patients are more psychologically inflexible and suggests that difficulty in weight management may be associated with the individual’s tendency to sacrifice long-term behavioral goals (e.g., healthy food choices and exercise) in the service of decreasing unpleasant and distressful internal experiences (e.g., food cravings and fatigue).

Moreover, this result is of great importance because BMI and adherence to medication are two relevant risk factors for cardiovascular disease. Notably, no between-group differences were detected using the AAQ-II, relative to both BMI and adherence. This result speaks to the incremental validity of CVD-AAQ and supports the usefulness of disease- and behavior-specific instruments for assessing psychological inflexibility.

Previous research suggests that psychological inflexibility plays an important role in the adoption and maintenance of health behaviors. Moreover, a preliminary investigation by Goodwin et al. (2012) found that a program to enhance psychological flexibility in a sample of cardiac patients effectively improved adherence to heart-healthy behaviors.

The current study is the first to evaluate the usefulness of a measure of psychological inflexibility specific to cardiovascular disease. The use of the CVD-AAQ may help identify patients who are more likely to be non-adherent. With further evaluation of the CVD-AAQ, it is possible that use of the instrument could help identify patients who are at risk for relapse or re-events. In an effort to begin such an evaluation, the CVD-AAQ will be employed to evaluate the mediational role of psychological flexibility in the context of a RCT comparing a brief ACT-based intervention to usual secondary prevention care of coronary heart disease (Spatola et al., 2014).

However, there are several limitations to the current study and further investigation is needed before this measure can be confidently used to assess the constructs of psychological inflexibility. Firstly, further tests of reliability and validity in other countries would ensure that the psychometric properties of this measure are well established and replicable. We considered running a confirmatory factor analysis in order to examine method variance, but since the initial one-factor solution which led to the development of the instrument was based on the same sample, we were concerned that any findings might be due to capitalization on chance, rather than true variance. Thus, we decided to not run this analysis until another sample could be obtained. Our recommendation is that future studies of the CVD-AAQ with other samples include a confirmatory factor analysis that allows for examination of whether the single factor solution found in our EFA is a better fit for the data than a two-factor solution. Secondly, the inclusion of a broader range of measures would be of particular utility for a deeper understanding of CVD-AAQ validity, with particular attention to be paid to other psychological inflexibility measures such as the Cognitive Fusion Questionnaire (Gillanders et al., 2014). Finally, longitudinal studies are needed in order to clarify the association between the CVD-specific psychological inflexibility and patient ability to adopt or maintain a heart-healthy lifestyle.

## Conflict of Interest Statement

The authors declare that the research was conducted in the absence of any commercial or financial relationships that could be construed as a potential conflict of interest.
